# Amorphous, Carbonated Calcium Phosphate and Biopolymer-Composite-Coated Si_3_N_4_/MWCNTs as Potential Novel Implant Materials

**DOI:** 10.3390/nano14030279

**Published:** 2024-01-29

**Authors:** Monika Furko, Rainer Detsch, Zsolt E. Horváth, Katalin Balázsi, Aldo R. Boccaccini, Csaba Balázsi

**Affiliations:** 1 Centre for Energy Research, HUN-REN, 1121, Konkoly-Thege Road 29-33, 1121 Budapest, Hungary; horvath.zsolt.endre@ek.hun-ren.hu (Z.E.H.); balazsi.katalin@ek-cer.hu (K.B.); 2 Institute of Biomaterials, Department of Materials Science and Engineering, University of Erlangen-Nuremberg, Cauer Str. 6, 91058 Erlangen, Germany; rainer.detsch@fau.de (R.D.); aldo.boccaccini@fau.de (A.R.B.)

**Keywords:** Si_3_N_4_, MWCNT, amorphous calcium phosphate, biopolymers, biocomposites

## Abstract

A biodegradable amorphous carbonated calcium phosphate (caCP)-incorporated polycaprolactone (PCL) composite layer was successfully deposited by a spin coater. In this specific coating, the PCL acts as a bioadhesive, since it provides a better adherence of the coatings to the substrate compared to powder coatings. The caCP–PCL coatings were deposited and formed thin layers on the surface of a Si_3_N_4_–3 wt% MWCNT (multiwalled carbon nanotube) substrate, which is an emerging type of implant material in the biomedical field. The composite coatings were examined regarding their morphology, structure and biological performance. The biocompatibility of the samples was tested in vitro with MC3T3-E1 preosteoblast cells. Owing to the caCP–PCL thin layer, the cell viability values were considerably increased compared to the substrate material. The ALP and LDH tests showed numerous living cells on the investrigated coatings. The morphology of the MC3T3-E1 cells was examined by fluorescent staining (calcein and DAPI) and scanning electron microscopy, both of which revealed a well-spread, adhered and confluent monolayer of cells. All performed biocompatibility tests were positive and indicated the applicability of the deposited thin composite layers as possible candidates for orthopaedic implants for an extended period.

## 1. Introduction

It is well-acknowledged that silicon nitride ceramic material has outstanding characteristics, such as chemical, biological and physical characteristics, and can be suitably shaped and produced for various medical applications. It has excellent mechanical features, as well as simultaneous osteogenic and antibacterial effects, which have been reported in some papers [1,2]. These properties make this material a good candidate for different implants. Generally, the micro- and nanostructure of the ceramic materials are strongly dependent on the preparation parameters and determine their final strength and performance [3].

However, the pristine silicon nitride ceramic has an intrinsic brittleness, which can place some limitations on its applicability. In order to enhance the mechanical properties of silicon nitride ceramics to make them more reliable, the structure of the base Si_3_N_4_ can be altered and adjusted by applying a second phase; for example, with carbon nanotubes (CNTs) [4,5]. A research work [5] thoroughly investigated how the CNT addition into the silicon nitride matrix affected its mechanical properties. They concluded that the appropriate CNT content enhanced both the fracture toughness and the overall mechanical strength, and they revealed that the optimal carbon content was 3 wt%. The CNT addition in a low amount can provide better stiffness, strength and good thermal as well as electrical conductivity to the base Si_3_N_4_ ceramics. There are research works that have reported the improved performance and higher porosity of CNTs containing Si_3_N_4_ composites [6,7,8,9]. However, these reports also revealed that the even dispersion of CNTs in the Si_3_N_4_ matrix is still a problem to be solved, since the uneven dispersion and agglomeration of CNTs noticeably worsen the composites’ characteristics. So far, one of the most effective ways to advance the dispersion and solidification is high-speed ultrasonic mixing [10]. The increased milling time during the milling procedure also improved the dispersion of additives as well as the milling parameters, and had a great effect on the morphology and microstructure of the final composites [11,12]. 

To make the silicon nitride even more biocompatible, the best way is to deposit calcium phosphate-based ceramic–biopolymer composites onto their surface [13,14]. Thanks to this innovative surface modification, the osseointegration can be faster and fewer early implant failures might occur [14]. The depositing of calcium phosphate-based (CP) layers onto implant materials is an extremely examined area to date. It is well-known that CPs can be well-integrated into the human body and, in the meantime, also promote new bone formation and ingrowth, as well as improve the fixation strength [15,16]. Recent in vivo results have reported that the damaged bone and implant devices can connect faster with the CP intermediate layer both in orthopedic and dental implants [17]. Among the CP phases, the amorphous calcium phosphate’s (aCP) biodegradation rate is the fastest [18,19]. It can be considered as a precursor material of hydroxyapatite generation and has a huge role in the biomineralization route. Moreover, aCP possesses outstanding bioactive properties and a manageable biodegradation rate [20,21]. It reportedly increased the ALP activity, enhanced the cell adherence and improved cell growth and proliferation [22,23]. The ratio of Ca and P elements in human bone components varies between 1.37 and 1.87 compared to that of 1.67 in the hydroxyapatite phase [24]. Additionally, the carbonate phase is considered as the most abundant anionic group substitute (from 3 to 8 weight percentage) in natural bone components. The CO_3_^2−^ anionic group can be incorporated into the apatite phase by substituting PO_4_^3−^ and OH^−^ anionic groups to generate carbonate-containing calcium phosphate phases (caCPs) [25].

As a new scientific breakthrough, the above-described caCP particles can be incorporated into different types of biodegradable polymers. These CP–biopolymer composites have also gained huge interest as biomaterials in orthopedics, medical and biomedical engineering, as well as in pharmaceutics. Among the numerous organic and synthetic biopolymers, polycaprolactone (PCL) is a promising candidate as a matrix material. The PCL polymer is a synthetic, aliphatic polyester that has some beneficial features, such as biodegradability and biocompatibility. It has a high *hydrolyzable* rate in the human body and the generated byproducts from the degradation process are inert and easy to excrete from the organisms. Therefore, polycaprolactone can be beneficially applied in numerous biomedical fields; for example, drug-encapsulating matrices with a controlled release [26], 3D-printed scaffolds [27], resorbable surgical sutures [28] and even biodegradable coatings [29]. Theoretically, the PCL degrades in biological conditions by its ester linkage hydrolysis. Therefore, it is an excellent candidate as a coating on implantable medical implants/devices due to its slower decomposition rate compared to other commonly used biopolymers, such as PVP, PLA, cellulose or cellulose acetate. [30,31]. In earlier studies, it was also reported that the PCL can completely degrade between two and four years, which depends on the polymer’s molecular weight [32].

It is worth noting that the majority of published studies on CP–PCL composites have concentrated on scaffold preparation, while their potential as coatings has received limited attention. As an example, Chunyan et al. [33] recently reported PCL/HAp composite layer preparation using hydrothermal and dipping methods. In their case, the layer was employed for the corrosion protection of the magnesium alloy substrate. The results revealed the improved adherence of the composite layer to the substrate compared to the pure HAp powder coating. In addition, in their case, the PCL/HAp composite layer also exhibited an improved biocompatibility. The composite layer can also be deposited on other types of substrates, such as titanium and ceramics, to make them more biocompatible or even bioactive [34]. The commonly used layer preparation techniques include dip coating [35], spin coating [36], the in situ sol–gel process [37] or electrospinning [38,39]. In a relatively recent work, Montanez et al. [36] developed a novel biocomposite layer. The layers contained PCL, different CaP phases and also multiwalled carbon nanotubes in various ratios. The biocomposite was then deposited onto titanium alloy with spin coating. 

In our present work, we deposited highly biocompatible caCP–PCL thin layers using the spin-coating method and investigated the layer’s complex morphological and structural characteristics. We also focused on the biological performance of the coatings using a preosteoblast cell line. These types of experiments and coating structures are still not investigated or reported thoroughly in the scientific literature. As a novelty, we have integrated the benefits of caCP and PCL, which resulted in a more biocompatible and very thin coating with good adherence to the Si_3_N_4_–MWCNT substrates.

## 2. Materials and Methods

### 2.1. Si_3_N_4_/3wt% MWCNT Substrate Preparation

The Si_3_N_4_/3wt% MWCNT composites were prepared with hot isostatic pressing (HIP) using oxidized α-Si_3_N_4_ powders and sintering additives. The starting powders included α-Si_3_N_4_ powder (Ube, SN-ESP) and multiwalled carbon nanotubes (MWCNTs) that were prepared with the catalytic chemical vapor deposition (CCVD) method, as described in [40]. The powder mixtures were oxidized at 1000 °C in ambient air conditions for 20 h. Then, the powder was sintered at 1700 °C for 3 h applying 20 MPa and N_2_ gas. The α-Si_3_N_4_ phase transformed into the β-Si_3_N_4_ phase during this process. The process is described in detail in reference [3]. The samples for further characterization were prepared in cubes with dimensions of 5 mm × 5 mm × 3 mm.

### 2.2. Preparation of Carbonated Amorphous Calcium Phosphate Nanopowder 

The suspension of caCP was prepared by wet chemical precipitation by dissolving calcium acetate (Ca(C_2_H_3_O_2_)_2_, Acros Organics BV, Geel, Belgium 99%) and disodium hydrogen phosphate (Na_2_HPO_4_, VWR International Ltd., Radnor, PA, USA, AnalaR). The Ca/P ratio was 5:3. The suspensions’ pH value was kept at 11 with a calculated content of sodium carbonate (anhydrous, VWR International Ltd., Radnor, PA, USA ≥99.5% ACS) to gain carbonated caCP nanoparticles. During the preparation process, the suspensions were stirred vigorously over 4 h at around 1400 rpm. The formed precipitates were cleaned thoroughly with distilled water and kept in an oven for 4 h at 150 °C. The resulting white nanopowders were gathered and prepared for further characterization and composite preparation.

### 2.3. The caCP/PCL Thin-Layer Deposition Using a Spin Coater

Polycaprolactone (PCL, average M_w_ ~80,000, Sigma-Aldrich, St. Louis, MO, USA) biopolymer was applied as a bioadhesive. The PCL solution was dispersed onto the Si3N4–3%MWCT substrate surface by a spin coater (Chemat Technology Spin Coater KW-4A, Chemat Scientific Inc., Northridge, CA, USA). The biopolymer was dissolved in dichloromethane (DCM, ACS reagent, ≥99.5%, Merck KGaA, Darmstadt, Germany) in a 10 *w*/*v*% concentration. In order to gain caCP particles containing PCL thin coatings, a 5 *w*/*v*% caCP/DCM suspension was prepared; then, the initial pure 10 *w*/*v*% polymer solution and the 5% (*w*/*v*) caCP/DCM suspension were mixed in a 2:1 ratio. The resulting white suspension then was pipetted onto the implant’s surface. The spin coating process was carried out as follows: first, the spin rate was 300 rpm to obtain an even and proper suspension distribution. Second, the spin rate was 1000 rpm to obtain a completely evaporated solvent. The pure PCL coating was prepared with the 10 *w*/*v*% polymer solution with the same parameters. 

### 2.4. Characterization Methods

#### 2.4.1. Morphological Characterizations by SEM and TEM Measurements

The morphology of the caCP particles, the polycaprolactone coating, the caCP–PCL coating as well as the seeded MC3T3-E1 cells on all samples were studied with a scanning electron microscope (SEM, Thermo Scientific, Scios2, Waltham, MA, USA). 

Transmission electron microscopy (TEM) evaluations were done with an FEI-Themis 200 G3 transmission electron microscope and a CS-corrected objective lens. EDS spectra were also recorded to determine the elemental ratios by a molybdenum grid. The Themis (Thermo Fisher, Waltham, MA, USA) TEM was used for the electron diffraction patterns. The Velox software with a 4k Ceta camera (Thermo Fisher) was used to record the SAED patterns. 

#### 2.4.2. Structural Characterizations by X-ray Diffractometry

The different phases in the samples were determined with XRD. The equipment included a Bruker AXS D8 Discover using a Cu Kα radiation source (λ = 0.154 nm) with a Göbel mirror and a scintillation detector (Bruker AXS, Karlsruhe, Germany). The crystalline phases were identified with Diffrac.Eva software. 

#### 2.4.3. FT-IR and Raman Measurements

The FT-IR was performed by the attenuated total reflection (ATR) technique. The equipment was a Varian Scimitar 2000 FT-IR spectrometer (SPW Industrial, Laguna Hills, CA, USA), and the detector was mercury–cadmium–telluride (MCT) fitted by a single-reflection diamond ATR unit (Specac Ltd., Orpington, UK). The spectra were recorded with a 4 cm^−1^ nominal resolution. The ATR corrections were applied before the evaluation. 

The Bio-Rad Digilab FT-Raman spectrometer (Triadscentific Inc., Manasquan, NJ, USA) with a Spectra-Physics Nd-YAG-laser (1064 nm) and a Ge detector was applied to record and evaluate the Raman spectra of all samples. The excitation laser power was 500 mW. Backscattered geometry was used with a 4 cm^−1^ nominal resolution during all measurements.

### 2.5. Biocompatibility Characterizations of the Prepared Samples and Composite Layers

All the samples were thoroughly studied and evaluated regarding their biocompatibility. The cell viability was assessed on the MC3T3-E1 preosteoblast cell line using the WST-8 assay (Sigma-Aldrich) after a cell culture of 3 days and 1 week with an Elisa plate reader (PHOmo, Anthos Mikrosysteme GmbH, Friesoythe, Germany) at 450 nm.

Lactate dehydrogenase (LDH) activity tests were performed to determine the amount of adhered cells on all samples. A commercial LDH-activity quantification kit was utilized in these measurements. The Elisa plate reader was also used at 490 nm and 690 nm.

The alkaline phosphatase (ALP) activity level was assessed after 3 days and 1 week of cell culture on all samples. The final solutions were measured at 405 nm and 690 nm with an Elisa plate reader. 

The cell distribution and morphology were also thoroughly studied by a fluorescent optical microscope (Axio Scope A.1, Carl Zeiss Microscopy Deutshland GmbH, Oberkochen, Germany). The seeded cells were stained with calcein and DAPI stains. The micromorphology and shapes of the seeded cells were further assessed by SEM measurements.

All the performed biocompatibility tests, cell culture procedures and standard protocols can be found in a more detailed description and an extended form in our previous study [41]

### 2.6. Statistical Examination

The statistical evaluation of the samples was assessed by a one-way analysis of variance (ANOVA, Origin 2021, OriginLab Corporations, USA) and by a Tukey test. The level of statistical significance was set to a *p*-value of 0.05. *p*-values below 0.05 (* *p* < 0.05) were statistically significant; below 0.01, they were statistically highly significant (** *p* < 0.01). The measurements were repeated six times (N = 6). 

## 3. Results and Discussion

### 3.1. Morphological Examination of the Substrate Starting Powder, the caCP Particles, the Polycaprolactone Polymer Coating as well as the caPC–PCL Thin Coating

**Figure 1:**
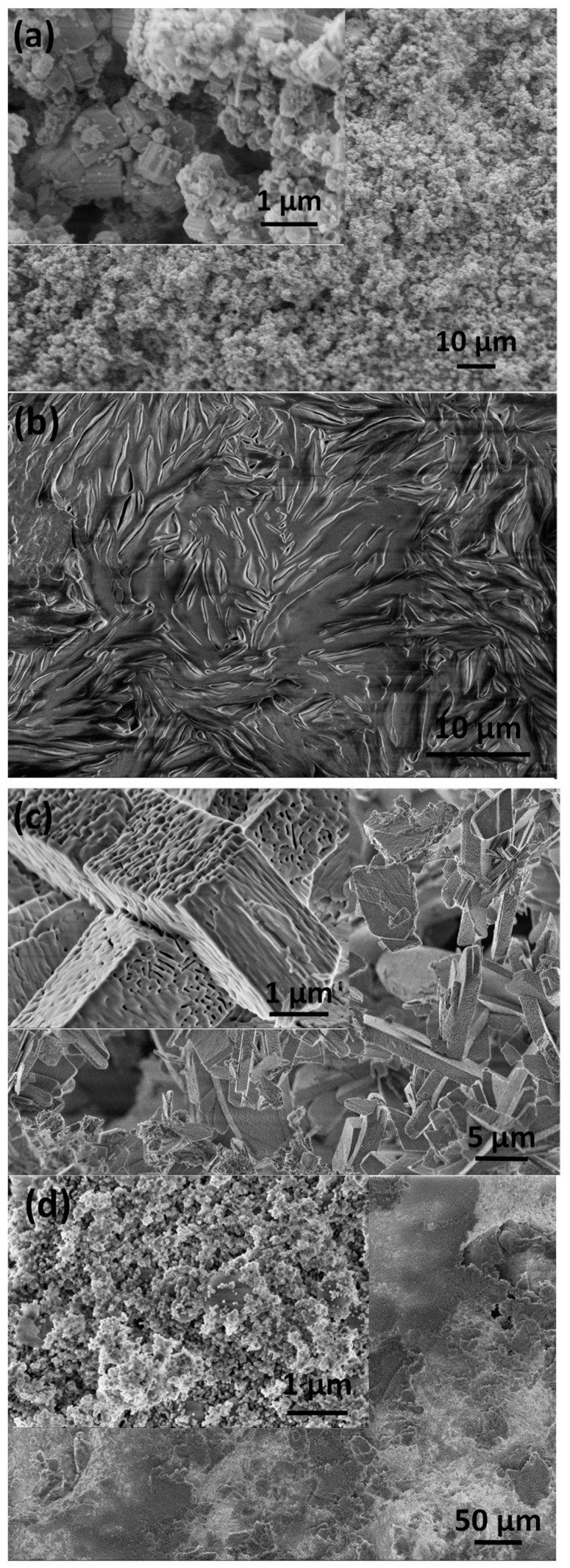
SEM image of the Si_3_N_4_–3 wt% MWCNT substrate powder before the sintering (**a**) of the pure PCL layer, (**b**) of the caCP nanopowder particles (**c**) and the caCP–PCL coating (**d**).

The morphologies of the prepared samples and thin layers were performed with SEM measurements. It can be observed that the substrate material consists of particles in two well-distinguished shapes. The Si_3_N_4_ particles are hexagonal-shaped columns in micrometer size, while the additive MWCNT particles are very thin platelets with a high aspect ratio in nanometer size (Figure 1a). In our case, the MWCNT particles were 0.3–0.4 mm in length and with a thickness of approximately 10 nm; moreover, the platelets can increase the porosity of the matrix [42]. The spin-coated pure PCL coating showed an amorphous, smooth morphology and a ripple texture (Figure 1b). The caCP particles, on the other hand, have a unique structure. As is visible, the small, needle-like caCP particles are agglomerated and form larger cuboid blocks with a high porosity. The sizes of the separated blocks are around 1 µm in thickness and roughly 3–6 µm in length (Figure 1c). The texture of these blocks seems highly porous, containing many inner holes which resemble the natural bones. This structure is also very beneficial for bone cell adherence and can advance the growth of cells [43,44,45]. 

The caCP–PCL thin coating, deposited with the same preparation parameters, has a noticeably different micro- and nanomorphology compared to the powder and the pure PCL (see Figure 1d). It can be observed that, in this case, the caCP particles do not form larger porous blocks, but rather small globular-shaped particles in nanometer size, and are incorporated into the large biopolymer grains, which are mainly in large plate shapes. The smaller and globular-shaped caCP particles adhered to the PCL plates in separate areas. 

**Figure 2:**
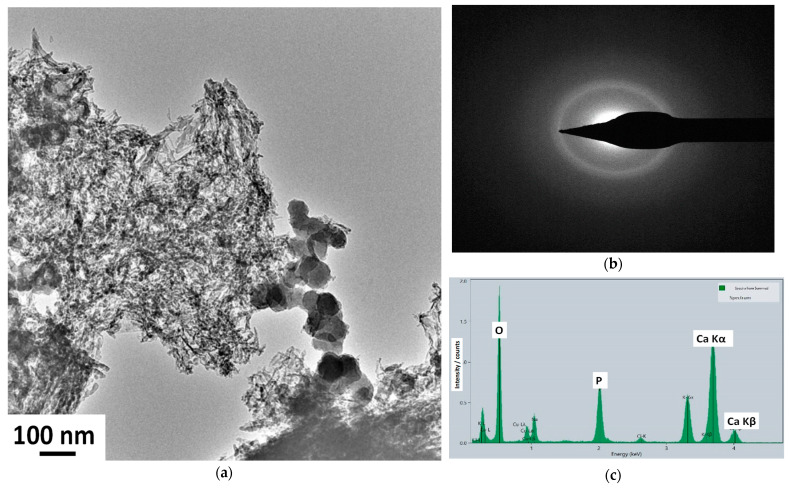
Low magnification TEM image (**a**), SAED pattern (**b**), and EDS measurement on the caCP powder (**c**). The electrons were collected from a large surface area.

To further analyze the nanostructure of the caCP powder, and to obtain a deeper insight, TEM–EDS measurements were also performed (Figure 2). 

According to the bright-field images, the sample consists of needle-like and spheric-shaped particles of around 30–70 nm in size. The SAED pattern of the sample confirmed its amorphous structure since, as is visible, a broad and diffused diffraction ring appears, which is characteristic of the amorphous structure. In this case, the SAED pattern was collected from a relatively large area of 400 nm, providing an averaged structural characteristic of the particles. The lack of distinct diffraction rings could indicate that the sample is nanocrystalline with highly disordered particles, or is amorphous.

Table 1 represents the element content of the caCP nanopowder measured by the TEM–EDS measurement. 

The obtained averaged Ca/P elemental ratio from the EDS measurements was 1.98, which is very similar to other reported ratios measured in natural bones [46]. Moreover, similar Ca/P ratios were also found in various ACP phases, as reported in other research works [47]. 

Some review papers [48,49] also described that the Ca/P ratios in amorphous CaPs change from 1.2 to 2.2, but another research work reported a Ca:P ratio of over 2 in the case of natural bones [46]. 

### 3.2. Structural Characterization of the Samples

The phase composition, as well as the micro- and nanostructure, of all samples were further studied by X-ray diffraction measurements, as well as FT-IR and Raman spectroscopy. 

**Figure 3:**
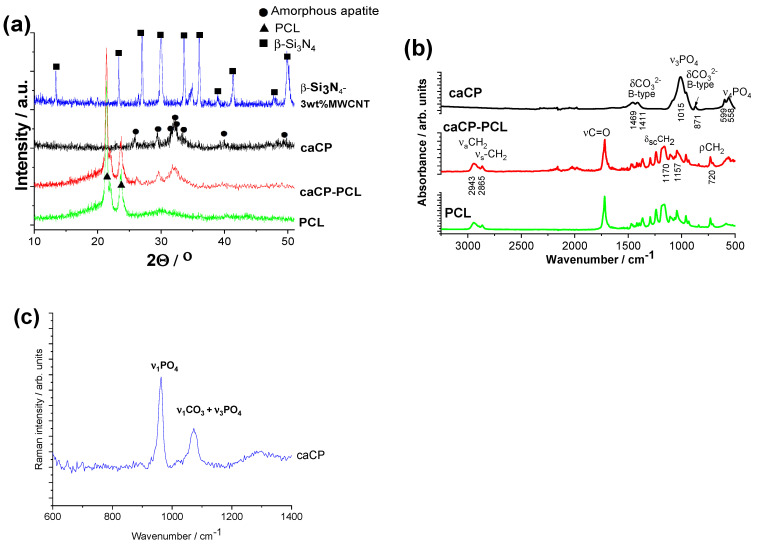
XRD measurements (**a**), FT-IR measurements (**b**), and Raman spectra (**c**) recorded on the investigated samples.

The X-ray diffraction of the substrate material has the characteristic peaks of the main phase of β-Si_3_N_4_ (JCPDS PDF-00-33-1160) (Figure 3a). There are no visible peaks on the XRD for the MWCNTs because of their low concentration in the sample matrix and the detection limit. The XRD pattern of the caCP powder demonstrates the strongest characteristic peaks of nanocrystalline or amorphous apatite at 2Θ = 31.7°, 32.2° and 32.9°, along with other minor peaks around 26°, 29° and 39°, respectively (JCPDS 01-086-1199). This pattern structure was also measured in other research works [50]. The spectrum in this case exhibits broad and merged peaks that support its nanocrystalline or amorphous features, or even the appearance of small, highly disordered nanoparticles [51]. Moreover, the presence of hydrogen phosphate, dihydrogen phosphate, carbonate and hydrogen carbonate anionic groups in the amorphous apatite also causes line broadening, as was written in an early study [52]. The XRD diffraction spectra of the PCL polymer as well as the caCP–PCL coating show the most intense peaks at 2Θ of 21.3° and 23.6°, which corresponds to the polycaprolactone according to JCPDS PDF 96-720-5590. However, the XRD pattern of the caCP–PCL has some additional peaks at 2Θ of 25° and 33°, which are also characteristic of the apatite phase.

Figure 3b represents the FT-IR measurements on different samples. The spectrum of caCP confirms that the powder is calcium phosphate or apatite and contains carbonate groups. The peaks at 1411 and 1469 cm^−1^ (ν3) stem from the carbonate anionic group stretching vibrations, while the weak peak at 871 cm^−1^ (ν2) demonstrates that the PO_4_^3−^ was partially replaced by CO_3_^2−^, resulting in a B-type carbonated apatite. The spectrum also has the peaks of the ν_3_PO_4_^3−^ group at 1015 cm^−1^ and the P-O antisymmetric splitting bond (ν4) at 558 and 599 cm^−1^. Hong et al. [53] also studied the carbonate amount in natural amorphous apatites, and it was also confirmed that the concentration of HPO_4_^2−^ ions showed a decreasing tendency with age in human bones, which can be related to the increase in the carbonate concentration [54]. The bioapatites usually incorporate carbonate as a prevalent substitute, generally from 2 to around 8 wt%, which can be dependent on the source material, such as bones, teeth, age and even species. 

The FT-IR spectra of the polycaprolactone and the caCP–PCL coating are noticeably different. For the PCL biopolymer, the most intense bands are connected to the methylene group, which is the symmetric and asymmetric stretching modes of CH_2_ at 2943 and 2865 cm^−1^. The CH_2_ scissoring band is at 1470 cm^−1^ and its rocking band is at 720 cm^−1^. The C=O stretching mode is present at 1720 cm^−1^ for the bands of the ester functional groups, while symmetric and asymmetric C−O−C stretching mode peaks are visible at 1240 and 1163 cm^−1^ [55].

For the composite layer, the characteristic absorption peaks are related to the polycaprolactone, but some extra weak bands also appear at the 500 to 600 cm^−1^ region, which is the P-O antisymmetric splitting bond (ν4) and peaks connected to ν_3_PO_4_^3−^ anions at 1015 cm^−1^. These results were also reported by Medeiros et al. [56]. They developed a PCL-based nanocomposite with graphene oxide containing hydroxyapatite. In this specific work, the content of HAp was kept at 20 wt%, while the graphene oxide concentration was varied in the samples; even so, they received very similar results from both the X-ray diffraction and the infrared spectroscopy measurements to our present achievements.

The Raman spectrum was also recorded on caCP powder (Figure 3c) to further prove the carbonated content in the apatite structure. The spectrum reveals the characteristic spectra of apatite with carbonate content. The most intensive band of the ν_1_PO_4_ vibration was at 959 cm^−1^ [55]. The specific band at 1072 cm^−1^ can be related to the B-type carbonate group when CO_3_^2−^ is substituting for PO_4_^3−^, as well as the ν_3_PO_4_ vibrations. According to the recorded spectra, the formation of B-type carbonated apatite is well-proven.

### 3.3. Biocompatibility Measurements In Vitro

#### 3.3.1. Viability of Preosteoblast Cells on Different Samples 

In our work, the Cell Counting Kit-8 (CCK-8) was used as a convenient assay, which utilizes water-soluble tetrazolium 2-(2-methoxy-4-nitrophenyl)-3-(4-nitrophenyl)-5-(2,4-disulfophenyl)-2H-tetrazolium salt (WST-8). In theory, oxidoreductase enzymes reduce the WST-8 reagent within the cells to generate a yellow formazan. The concentration of the yellow-colored formazan in the cells correlates to the amount of live cells. 

**Figure 4:**
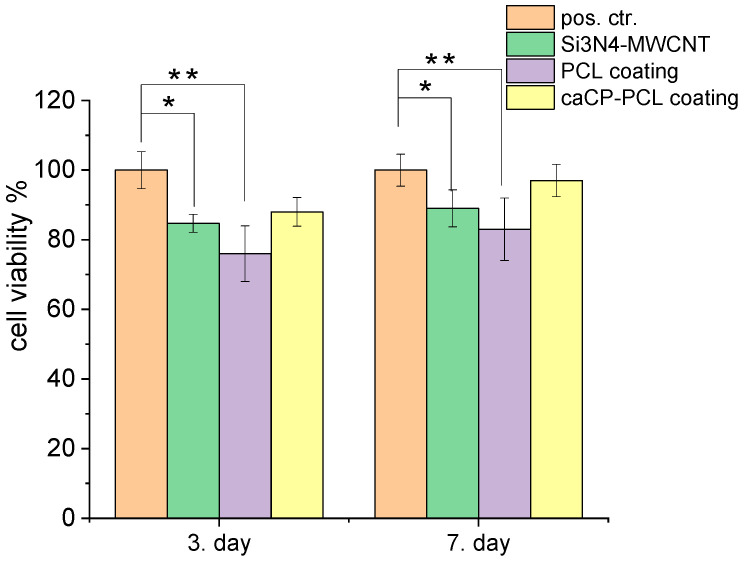
Cell viability percentage related to the positive control ((±SD) positive control (REF = 100%): only the MC3T3-E1 cells were grown in the well plates for 3 and 7 days. Statistical significance level was measured by *p*-values: * *p* < 0.05 means the differences are statistically significant; ** *p* < 0.01 means the differences are statistically highly significant. The graphs show the mean value ± standard deviation of six replicates of each sample group.

The cell viability studies clearly revealed a high level of cell viability, around 84–98% on all types of samples, compared to the positive control (see in Figure 4). The graph shows that the difference in cell viability values between the control and substrate was statistically significant, and between the control and the PCL coating, the difference was statistically highly significant after both 3 days and 1 week of cell culture. There is a very slight and nonsignificant difference between the coated and the uncoated substrate; in some cases, the coating caused a 3–8% increase in cell viability. Moreover, it can be also observed that the cell viability, and thus the biocompatibility of the samples, increased with the increased culture time. The most biocompatible coating in this case was the Si_3_N_4_/3wt% MWCNT/caCP–PCL sample, showing a 97% cell viability, while the lowest biocompatibility belonged to the PCL coating (83% after a one-week cell culture), which can be ascribed to the hydrophilic nature of this biopolymer. However, this hydrophilic nature can be overcome with the incorporation of apatite, which is clearly shown in the cell viability values. 

#### 3.3.2. Cell Concentration on Samples and Lactate Dehydrogenase (LDH) Tests

The level of LDH activity correlates to the number of adhered cells onto the samples’ surface, and the alkaline phosphatase enzyme generation is a useful indicator of viable cells and can show the appearance of osteoblast cells and new bone generation.

**Figure 5:**
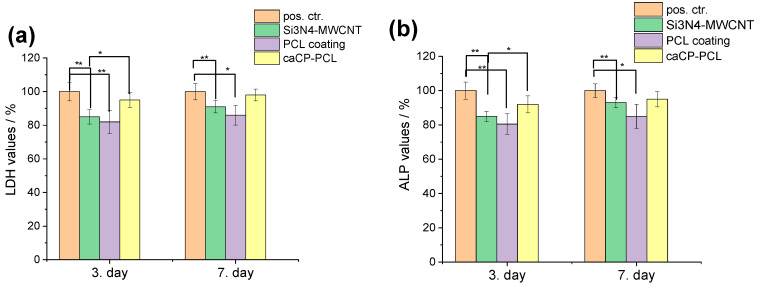
Lactate dehydrogenase (**a**) and alkaline phosphatase (**b**) activities (mean percentages ± standard deviations) of the investigated samples. Positive control: only the MC3T3-E1 cells were grown in well plates for 3 and 7 days. Samples were measured in 6 replicates. Lysis buffer was used to induce cell death. The statistical significance level was determined at *p* values: * *p <* 0.05 (significant); ** *p <* 0.01 (highly significant).

The values of lactate dehydrogenase activity (Figure 5a) proved the presence of live cells on all types of samples. It is visible that the amount of living cells is the highest in the case of the caCP–PCL-composite-coated substrate material, while the lowest values were obtained on the PCL coating. This result agrees well with the cell viability measurements. The alkaline phosphatase activity level (Figure 5b) demonstrated a very similar trend to that of the LDH measurements; thus, the highest ALP values were obtained on the caCP–PCL composite layer. The statistical evaluation of these tests (ALP and LDH) provided a quite identical result. The ALP and LDH values were statistically highly significant (** *p <* 0.01) between the positive control and the Si_3_N_4_/3wt% MWCNT and the PCL layer. The difference between the values of the Si_3_N_4_/3wt% MWCNT and the caCP–PCL composite layer was also statistically significant (* *p <* 0.05) on the third day of the cell culture; however, after one week, the difference was not more significant. This could mean that the MC3T3-E1 cells required more time to adhere to the surface of the pure PCL thin layer and differentiate because the environment and conditions might be less beneficial than in the case of the caCP-containing biopolymer layers. As a conclusion, the LDH and ALP activity levels were the lowest on the PCL coating. The Si_3_N_4_/3wt% MWCNT substrate was appropriately biocompatible and the caCP addition to the biopolymer matrix noticeably advanced the cell attachment. The obtained results here agree well with other research works, in which the amorphous CaP particles and different CaP–biopolymer composites were studied and characterized concerning their boosted osteogenic level [57,58,59,60,61,62,63]. 

#### 3.3.3. Morphological Evaluation of the MC3T3-E1 Preosteoblast Cells

The cells grown on the investigated samples were stained with calcein and DAPI following a strict protocol. The stainings were performed in order to study the cell morphology. The staining process was performed after 24 h of cell culture in a DMEM solution.

**Figure 6:**
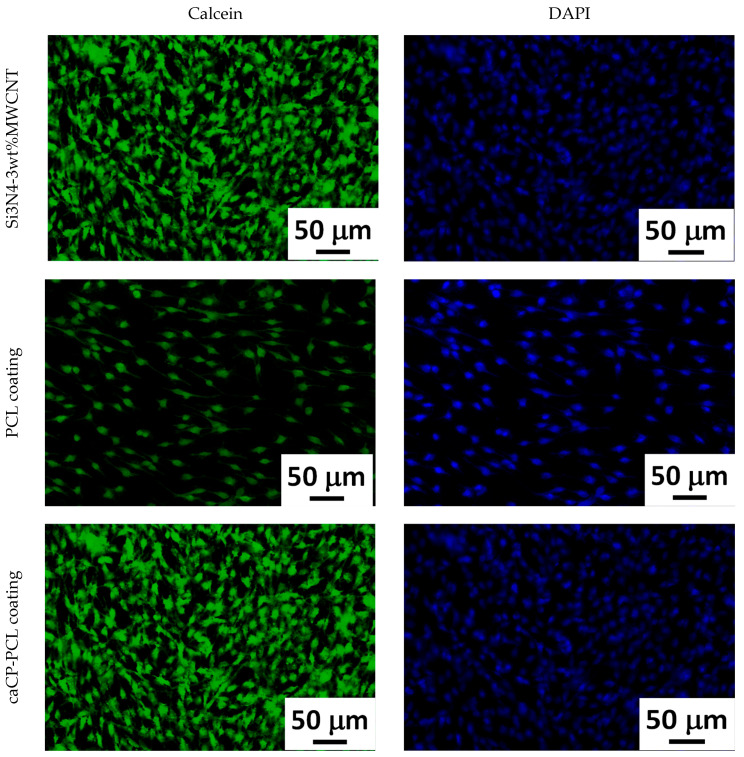
Calcein and DAPI staining of the MC3T3-E1 cells incubated on the studied samples for 24 h.

Figure 6 clearly reveals a large number of live cells on all investigated samples The cells were grown on the surface of the samples for 24 h. The cell concentration is apparently less on the pure PCL layer. This can be explained by the fact that the polycaprolactone is a hydrophilic polymer in nature, which could impede the cell adherence. On the contrary, a very dense and even monolayer of cells can be observed on the surface of the Si_3_N_4_/3wt% MWCNT substrate, as well as on the caCP–PCL composite coating. The highest density can be seen on the surface of the composite layer. This also correlates very well with the results from the previous biocompatibility tests. It is noticeable that the amount of caCP particles within the biopolymer matrix significantly enhanced the biocompatibility, which was undoubtedly proven by the confluent and dense preosteoblast cell layer adhered and proliferated on the surface of the samples.

**Figure 7:**
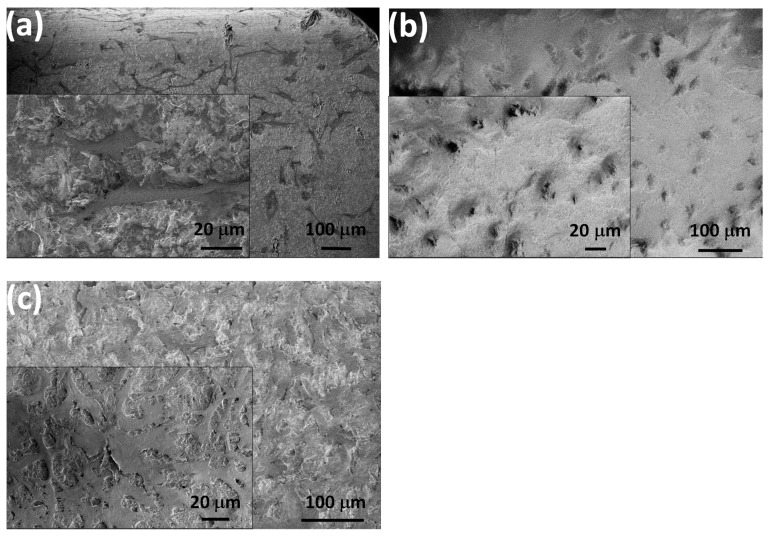
SEM images of the seeded MC3T3-E1 cells after 24 h of cell culture on the Si_3_N_4_/3wt% MWCNT substrate (**a**) on the PCL layer (**b**) and on the caCP–PCL composite layer (**c**).

In Figure 7, we show the morphological characteristics of the attached cells after 24 h of cell culture in the DMEM solution by performing SEM measurements. 

The MC3T3-E1 cells showed a normal morphology in all cases. The shape of the cells was polygonal, and they were widespread, which is the best growth shape of the cells. Moreover, the cells also demonstrated numerous protrusions and flattened extensions that grew and penetrated into the pores of the layers. The cellular extensions and protrusions proved to be properly attached and growing [64,65]. It can also be observed that the spread form of the cells on the caCP–PCL composite coating was more notable, and had more protrusions compared to the cells seeded on both the Si_3_N_4_/3wt% MWCNT sample and the PCL layer. 

We have to mention that, besides the numerous advantages of these composite coatings, there are some drawbacks too. These limitations include the poor reproducibility, the uncontrollable pore sizes and shapes as well as the confined pore interconnectivity. Proper porosity is crucial for good integration and for enhanced osteoconductivity. Another critical parameter is the layer thickness. According to the results of our extensive experiments we have found that very thin layers (2 μm or below) are ideal for application, while thicker layers are prone to detach. These drawbacks, however, can be eliminated by the optimization of the deposition process. The caCP–PCL composite layers are intrinsically biodegradable, but the specific way in which they decompose, the rate at which the metabolic products are eliminated and their impact on the human body are yet to be fully understood. The optimal implant coatings should meet all crucial conditions, such as a proper surface roughness, good adhesion, porosity, biocompatibility and controlled biodegradability. The caCP–PCL thin composites that we developed could have potential as coatings on different implant substrates, since they showed very promising morphological and biological characteristics.

## 4. Conclusions

Highly biocompatible, carbonated and amorphous calcium phosphate–polycaprolactone composite coatings were developed and deposited onto Si_3_N_4_/3wt% MWCNT substrate material by the spin-coating method. The MWCNT containing the Si_3_N_4_ ceramic is an innovative type of biomaterial with emerging applications in many biomedical fields. The purpose of the surface modification by the caCP–PCL coating was to make the base ceramic material even more bioactive. 

The morphological investigations revealed the PCL coating to be amorphous with a smooth and ripple texture. The caCP particles were in a small, needle-like form and agglomerated into larger cuboid blocks with a high porosity. On the contrary, the caCP–PCL composite coating deposited with the same route and parameters had a different micro- and nanostructure. The caCP particles were small, globular-shaped and did not form larger porous blocks. The particles were incorporated into the larger polymer particles. 

The TEM–EDS and XRD measurements proved the caCP powder to be amorphous apatite, while the FT-IR and Raman spectra proved the carbonate substitution in the CaP phase.

The in vitro studies revealed important results, since the cell viability tests proved that the largest amount of viable MC3T3-E1 cells were grown on the surface of the caCP–PCL composite layer compared to the silicon nitride substrate and the pure biopolymer layer after 3 and 7 days. The pure PCL coating was the least cytocompatible; however, the caCP powder incorporation into the PCL matrix significantly enhanced the in vitro biocompatibility of the samples. Analyses with the fluorescence microscope and the SEM similarly verified the large amount of live and well-spread MC3T3-E1 cells that formed a confluent and very dense monolayer. The cells also had numerous osteoblastic phenotype expressions. 

Overall, the present study clearly proved that the developed novel, spin-coated caCP–PCL thin layer is an advantageous surface modification for silicon nitride-based ceramic implants.

## Figures and Tables

**Table 1:** Mean percentages of the detected elements (±standard deviations) in the investigated caCP nanopowder (N = 6).

Element	Ca	P	C	O	Ca/P
Ratio (Wt.%)	26.86 ± 6.44	13.53 ± 3.18	16.21 ± 3.93	43.40 ± 5.14	1.98

## Data Availability

The data that support the findings of this study are available from the corresponding authors, M.F. or C.B., upon reasonable request.
